# Vitamin C increases 5-hydroxymethylcytosine level and inhibits the growth of bladder cancer

**DOI:** 10.1186/s13148-018-0527-7

**Published:** 2018-07-13

**Authors:** Ding Peng, Guangzhe Ge, Yanqing Gong, Yonghao Zhan, Shiming He, Bao Guan, Yifan Li, Ziying Xu, Han Hao, Zhisong He, Gengyan Xiong, Cuijian Zhang, Yue Shi, Yuanyuan Zhou, Weimin Ci, Xuesong Li, Liqun Zhou

**Affiliations:** 1 Department of Urology, Peking University First Hospital, Beijing, 100034 China; 2 Key Laboratory of Genomic and Precision Medicine, Beijing Institute of Genomics, Chinese Academy of Sciences, Beijing, 100101 China; 3 Institute of Urology, Peking University, Beijing, 100034 China; 4 National Urological Cancer Center, Beijing, 100034 China; 5 Urogenital Diseases (Male) Molecular Diagnosis and Treatment Center, Peking University, Beijing, 100034 China; 6 University of Chinese Academy of Sciences, Beijing, 100049 China

**Keywords:** Bladder cancer, 5-Hydroxymethylcytosine, Vitamin C, TET

## Abstract

**Background:**

5-Hydroxymethylcytosine (5hmC) is converted from 5-methylcytosine (5mC) by a group of enzymes termed ten-eleven translocation (TET) family dioxygenases. The loss of 5hmC has been identified as a hallmark of most types of cancer and is related to tumorigenesis and progression. However, the role of 5hmC in bladder cancer is seldom investigated. Vitamin C was recently reported to induce the generation of 5hmC by acting as a cofactor for TET dioxygenases. In this study, we explored the role of 5hmC in bladder cancer and the therapeutic efficacy of vitamin C in increasing the 5hmC pattern.

**Results:**

5hmC was decreased in bladder cancer samples and was related to patient overall survival. Genome-wide mapping of 5hmC in tumor tissues and vitamin C-treated bladder cancer cells revealed that 5hmC loss was enriched in cancer-related genes and that vitamin C treatment increased 5hmC levels correspondingly. Vitamin C treatment shifted the transcriptome and inhibited the malignant phenotypes associated with bladder cancer cells in both in vitro cell lines and in vivo xenografts.

**Conclusions:**

This study provided mechanistic insights regarding the 5hmC loss in bladder cancer and a rationale for exploring the therapeutic use of vitamin C as a potential epigenetic treatment for bladder cancer.

**Electronic supplementary material:**

The online version of this article (10.1186/s13148-018-0527-7) contains supplementary material, which is available to authorized users.

## Background

Bladder cancer is the most common cancer of the urogenital system, ranking sixth among all cancers and fourth in males [1, 2]. Despite the advancements in surgical techniques and chemotherapy, the outcome of bladder cancer is still poor, especially in patients with advanced and metastatic disease [3]. Therefore, there is a pressing need for novel biomarkers that can stratify the risk of mortality and provide potential therapeutic targets.

Alterations in DNA methylation are among the earliest and most common events in tumorigenesis [4]. Global loss and promoter-associated gains of DNA methylation (5-methylcytosine, 5mC) have been considered to be the hallmarks of cancers [5–7]. Recently, 5-hydroxymethylcytosine (5hmC) was discovered as a transformed form of 5mC via ten-eleven translocation (TET) enzymes in the demethylation cycle [8]. In a number of cancers, 5hmC has been observed to be remarkably decreased and associated with tumorigenesis, progression, and outcomes [9–12].

Several studies have also reported the loss of 5hmC in bladder cancer [13, 14]. However, the genome-wide profile and role of 5hmC in bladder cancer tumorigenesis, progression, and outcome have been seldom investigated. Vitamin C is a co-substrate of Fe (II)-2-oxoglutarate-dependent dioxygenases, including TETs [15]. Recently, vitamin C was reported to block leukemia progression and promote differentiation by enhancing 5hmC formation [16, 17].

Here, we compared the 5hmC genome-wide profiles between normal bladder and bladder cancer tissues and characterized the association between 5hmC and bladder cancer tumorigenesis, progression, and outcomes. We showed that vitamin C could increase 5hmC levels and inhibit malignant phenotypes in bladder cancer both in vitro and in vivo. Loss of 5hmC could be a novel biomarker and treatment target for bladder cancer.

## Results

### 5hmC level is an independent molecular marker of bladder cancer

**Fig. 1:**
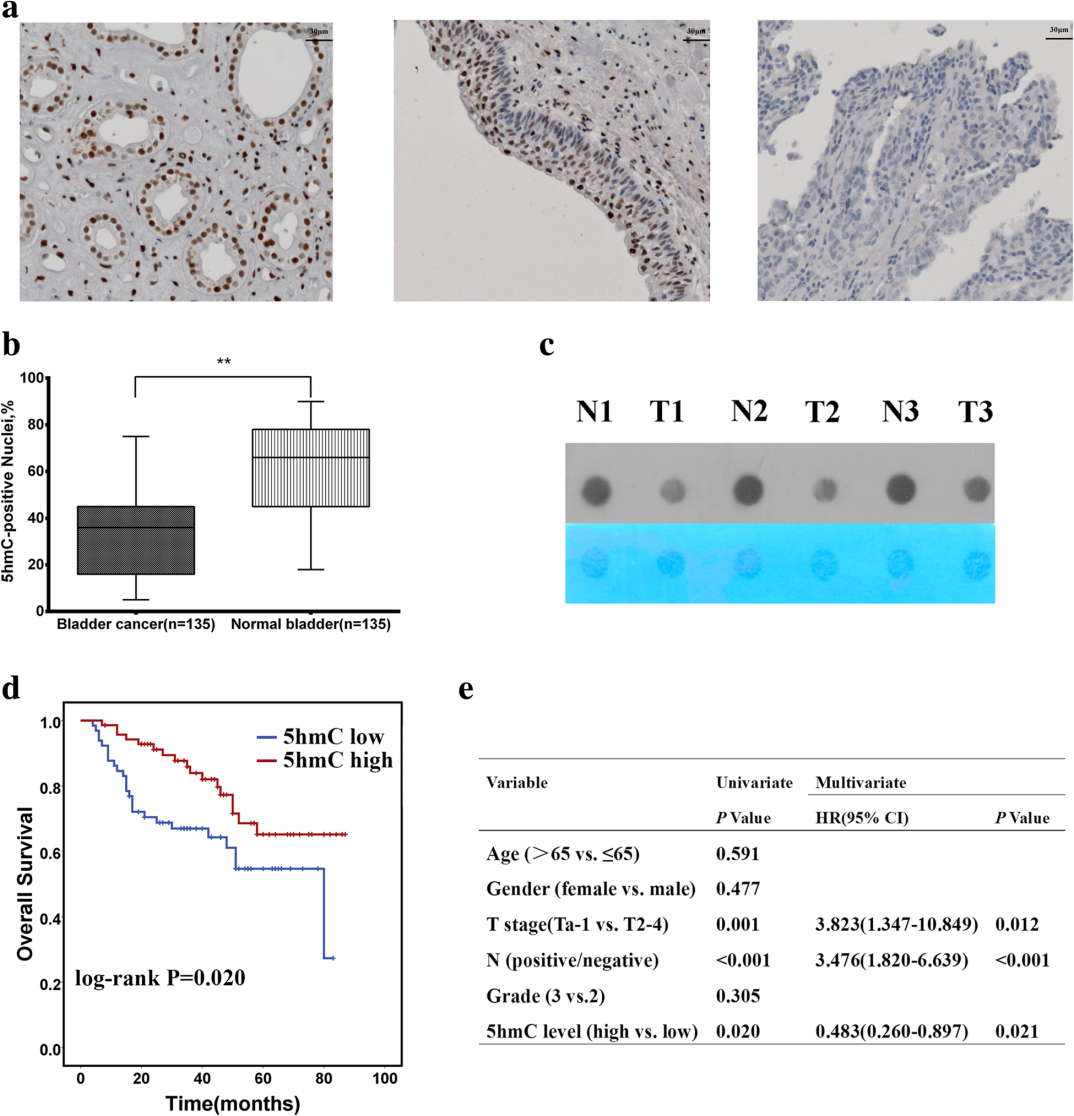
Loss of 5hmC is a hallmark of bladder cancer. **a** IHC staining of 5hmC in the positive control (normal kidney) and representative bladder cancer and normal bladder samples. Scale bar, 30 μm. **b** Analysis of 5hmC levels in bladder cancer and normal bladder samples represented by a 5hmC score. Statistical significance was determined by the Mann-Whitney *U* test. **c** Dot blot assay of 5hmC levels in normal bladder tissues relative to bladder cancer tissues. **d** Kaplan-Meier survival curves of bladder cancer patients with high and low 5hmC staining. *P* value was calculated by the log-rank test. **e** Multivariate Cox regression analyses of bladder cancer cases

We first detected 5hmC levels in normal bladder and bladder cancer tissues by immunohistochemistry (IHC) staining with formalin-fixed, paraffin-embedded tissue sections. The clinicopathological characteristics of 135 patients with bladder urothelial carcinoma are shown in Table 1. Consistent with recent findings in other types of cancers, normal bladder tissues showed strong nuclear 5hmC staining (*n* = 135), whereas bladder cancer showed a partial or complete loss of 5hmC (*n* = 135) (*P* < 0.05, Fig. 1a, b; normal renal tissue was used as a positive control). The loss of 5hmC was also confirmed by anti-5hmC antibody-based dot blot assay in matched bladder cancer and normal bladder tissues (Fig. 1c). A Kaplan-Meier log-rank test revealed that patients with higher 5hmC levels had significantly longer overall survival than patients with lower 5hmC levels (Fig. 1d). Further, univariate and multivariate Cox proportional hazard regression analyses showed that the 5hmC level in tumor tissues independently provided predictive power and that lower 5hmC levels correlated with shorter overall survival, as reflected by the hazard ratio of 0.483 (Fig. 1e), suggesting that the loss of 5hmC is critical for bladder cancer progression. Meanwhile, a lower 5hmC level was also associated with a higher tumor stage and lymphatic metastasis (Table 2).

**Table 1:** Clinicopathological characteristics of 135 patients with bladder urothelial carcinoma

Characteristics	Total *n* = 135
Age, years, median (IQR)	66 (56–74)
Gender	
female	23
male	112
Grade	
2	35
3	100
T stage	
Tis	3
T1	36
T2	39
T3	25
T4	32
N status	
Negative	110
Positive	25

*IQR* interquartile range

**Table 2:** Association between clinicopathological characteristics and 5hmC level of bladder urothelial carcinoma

Cohort characteristics	5hmc level		Chi-square test
	Low	High	*P* value
Grade			0.514
2	20	18	
3	45	52	
Age			0.412
< 65	27	34	
≥ 65	38	36	
T stage			0.010
Tis–T1	12	27	
T2–T4	53	43	
N stage			0.028
Negetive	47	62	
Positive	18	8	

### Genome-wide mapping of 5hmC in paired tumor and adjacent normal tissue

**Fig. 2:**
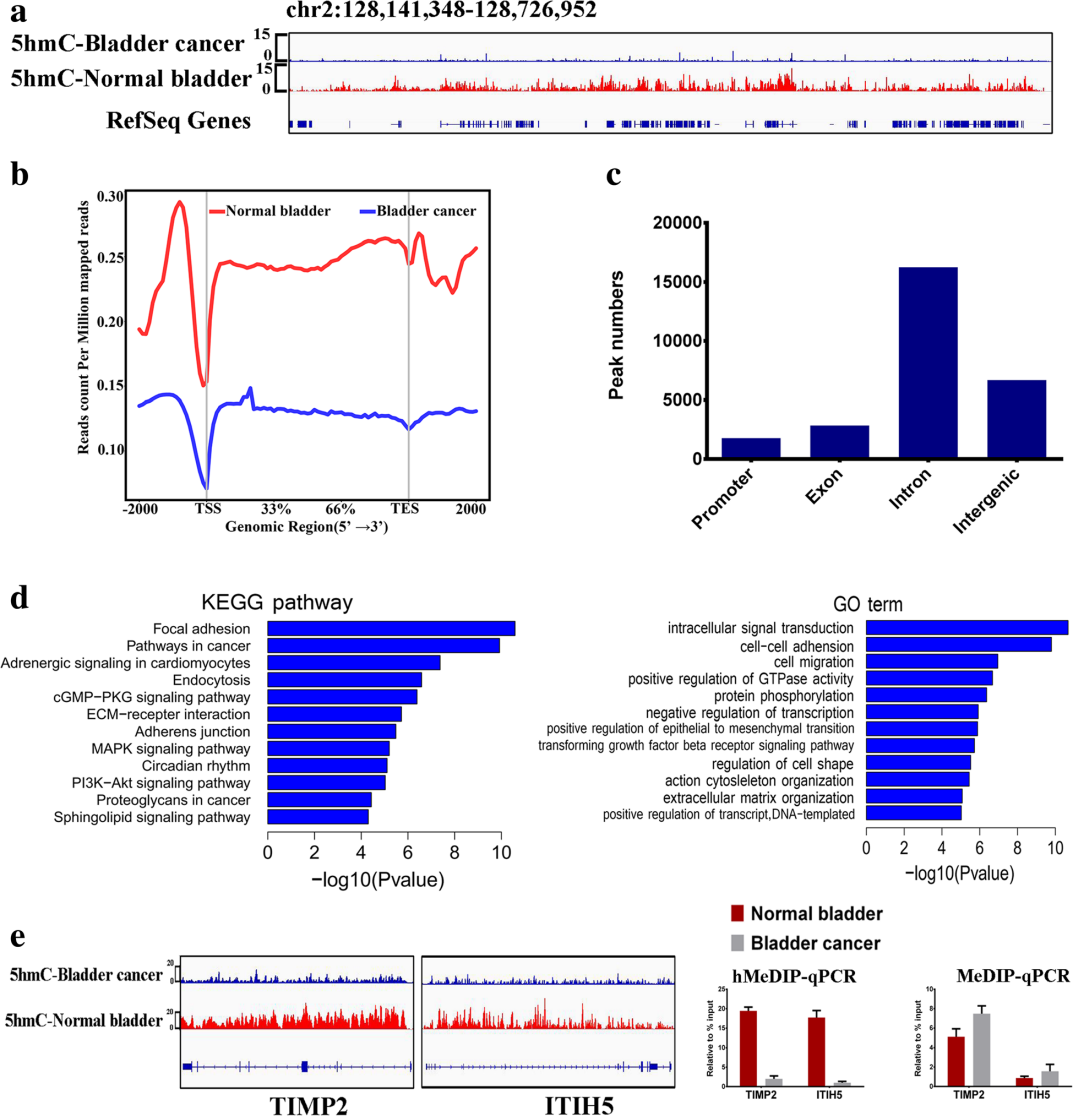
Genome-wide mapping of 5hmC in bladder cancer and normal bladder. **a** The distribution of 5hmC densities in the chr2:128,141,348-128,726,952 regions by hMeDIP-seq. RefSeq genes are shown at the bottom. **b** The average 5hmC levels in normal bladder and bladder cancer tissues across different gene-associated regions. **c** Significant 5hmC peak numbers in normal bladder and bladder cancer samples in different genomic regions. Promoters were defined as − 2k to + 2k relative to the TSS. **d** KEGG pathway and GO term analysis results for significant 5hmC peak-associated genes. **e** The hMeDIP-seq results (left) and hMeDIP-qPCR/MeDip-qPCR verifications of representative TIMP2 and ITIH5 genes

To explore whether 5hmC loss during bladder tumorigenesis was genome-wide or locus-specific, we used a hydroxymethylated DNA immunoprecipitation (hMeDIP) approach coupled with deep sequencing (hMeDIP-seq) to compare genome-wide changes in 5hmC between the normal bladder and bladder cancer tissue. We found that 5hmC is associated with gene-rich regions in the normal bladder genome and is relatively low in the bladder cancer genome (Fig. 2a). Importantly, we observed a significant decrease in 5hmC levels within the average gene and in the regions 2000 bp up- and downstream of the gene in bladder cancer tissue compared with normal bladder tissue (Fig. 2b). Using MACS software, we identified 27,565 5hmC peaks that were decreased in bladder cancer (FDR < 0.05), more than half of which were located either in exons (10.3%) or introns (58.9%) and 6.45% of which were located at the promoters (Fig. 2c). Using CEAS software, 27,565 5hmC peaks were mapped to 5843 genes. KEGG pathway enrichment and GO term analyses of the 5843 genes revealed that these genes are closely associated with various cancer-related pathways (Fig. 2d). As an example, the TIMP2 and ITIH5 genes showed decreased 5hmC in gene bodies in bladder cancer compared with the normal bladder samples, and hMeDip-qPCR/MeDip-qPCR verified the increase in 5hmC and a relative decrease in 5mC (Fig. 2e). Collectively, the loss of 5hmC occurred in multiple cancer-related genes during bladder carcinogenesis.

### Increasing 5hmC by vitamin C can inhibit the malignant phenotype of bladder cancer cells

**Fig. 3:**
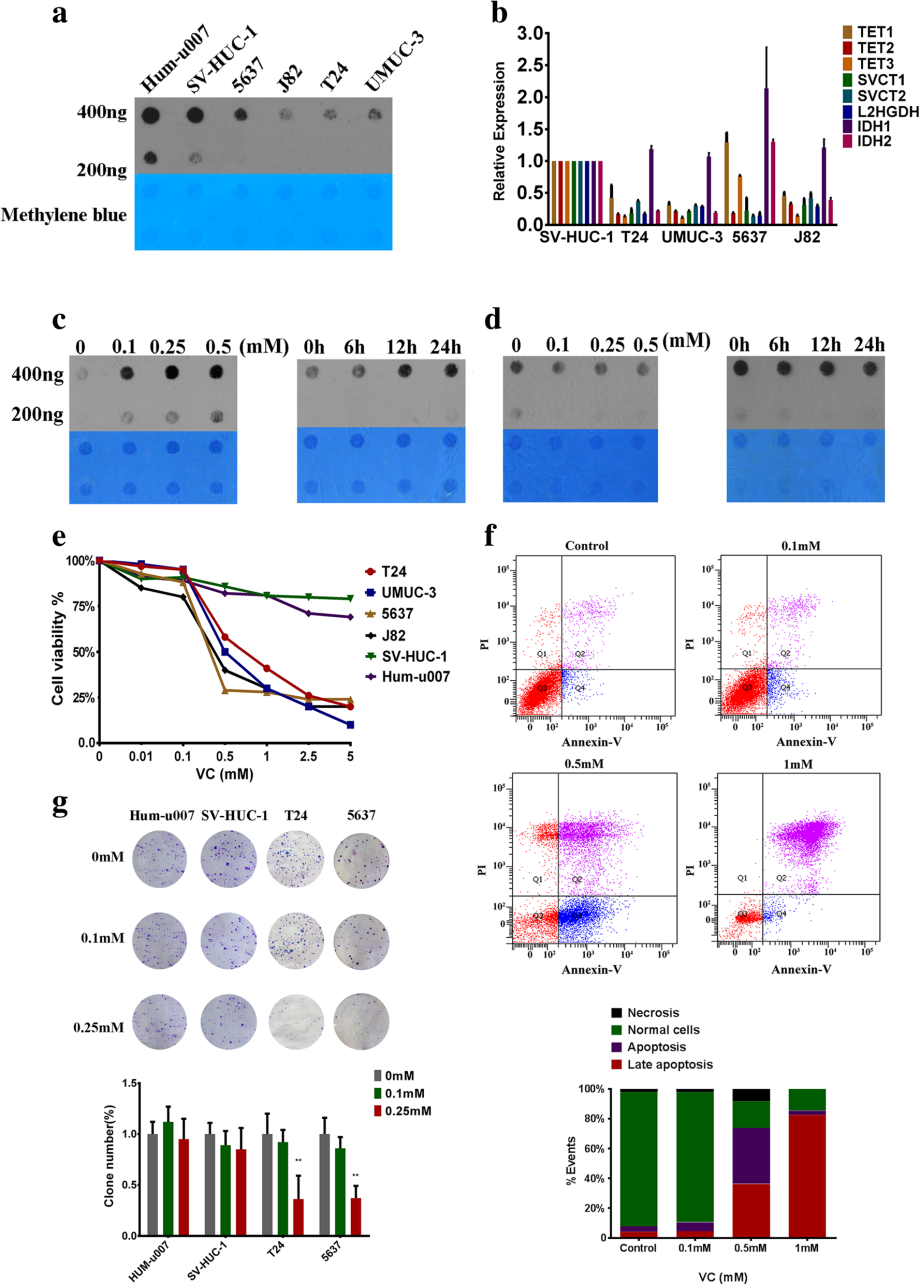
Vitamin C treatment increases 5hmC levels and decreases malignant phenotypes in bladder cancer cells in vitro. **a** Dot blot assay of 5hmC levels in normal bladder and cancer cell lines. **b** The relative transcription levels measured by RT-qPCR of 5hmC-related genes and SVCTs in normal bladder and bladder cancer cells. **c** Dot blot assay of 5hmC levels of T24 cells at varying concentrations and time periods with vitamin C. **d** Dot blot assay of 5mC levels of T24 cells at varying concentrations and time periods with vitamin C. **e** MTS assay of cell viability for normal bladder and cancer cell lines at varying concentrations with vitamin C. **f** Apoptosis assay of T24 cells at varying concentrations with vitamin C. **g** Clone formation assay for normal bladder and cancer cell lines at varying concentrations with vitamin C. Statistical significance was determined by the Mann-Whitney *U* test

We next detected the 5hmC levels in normal and bladder cancer cell lines. According to dot blot assay, 5hmC content was relatively high in nonmalignant cells (primary urothelial cells Hum-u007 and immortalized normal cells SV-HUC-1) but was low in bladder cancer cells (Fig. 3a). To explore the molecular mechanisms underlying 5hmC loss in bladder cancer, we also measured the expression of 5hmC-related enzymes (TET1, TET2, TET3, IDH1, IDH2, and L2HGDH) and vitamin C transporters (SVCT1 and SVCT2) by RT-qPCR and found that the expression of TET2, L2HGDH, and vitamin C transporters (SVCT1 and SVCT2) were relatively decreased in all bladder cancer cell lines (Fig. 3b). Vitamin C is capable of increasing 5hmC by acting as a cofactor of TET proteins. We then treated several bladder cancer cell lines with vitamin C at varying concentrations and time periods. As a result, vitamin C increased the 5hmC levels in T24 cells in a time- and concentration-dependent manner (Fig. 3c). Meanwhile, global 5mC levels were slightly decreased (Fig. 3d). J82 and 5637 cells showed similar results (Additional file 1: Figure S1B and C). Moreover, we observed similar effects with vitamin C, which increased 5hmC levels and inhibited cancer cell growth in renal cancer cells (Additional file 1: Figure S1D). We also measured relative TET1/2/3 expression levels after vitamin C treatment by RT-qPCR and found no significant changes (Additional file 2: Figure S2A). This result indicated that vitamin C increases 5hmC levels in bladder cancer cells by promoting the activity of TET enzymes rather than by increasing the expression levels. In the cell proliferation analysis by MTS assay at varying concentrations, vitamin C significantly inhibited bladder cancer cell proliferation at pharmacological concentrations (0.5 to 5 mM), although it was relatively less toxic to nonmalignant cells (Fig. 3e). High-dose vitamin C also induced significant apoptosis in T24 cells (Fig. 3f and Additional file 2: Figure S2B). Colony formation assays also demonstrated the inhibitory effects of vitamin C in bladder cancer cells relative to nonmalignant cells (Fig. 3g). It was reported that high-dose vitamin C can inhibit cancer cells by H_2_O_2_ production. To eliminate the influence of H_2_O_2_, we added 100 μg/ml catalase to block all H_2_O_2_ and found that the inhibition of high-dose vitamin C was partially rescued; however, vitamin C still had a suppression effect (Additional file 2: Figure S2C). In summary, high-dose vitamin C could directly induce growth arrest and apoptosis in bladder cancer cells, unlike low-dose vitamin C, which suppressed cancer cell growth in an H_2_O_2_-independent manner that included 5hmC restoration.

**Fig. 4:**
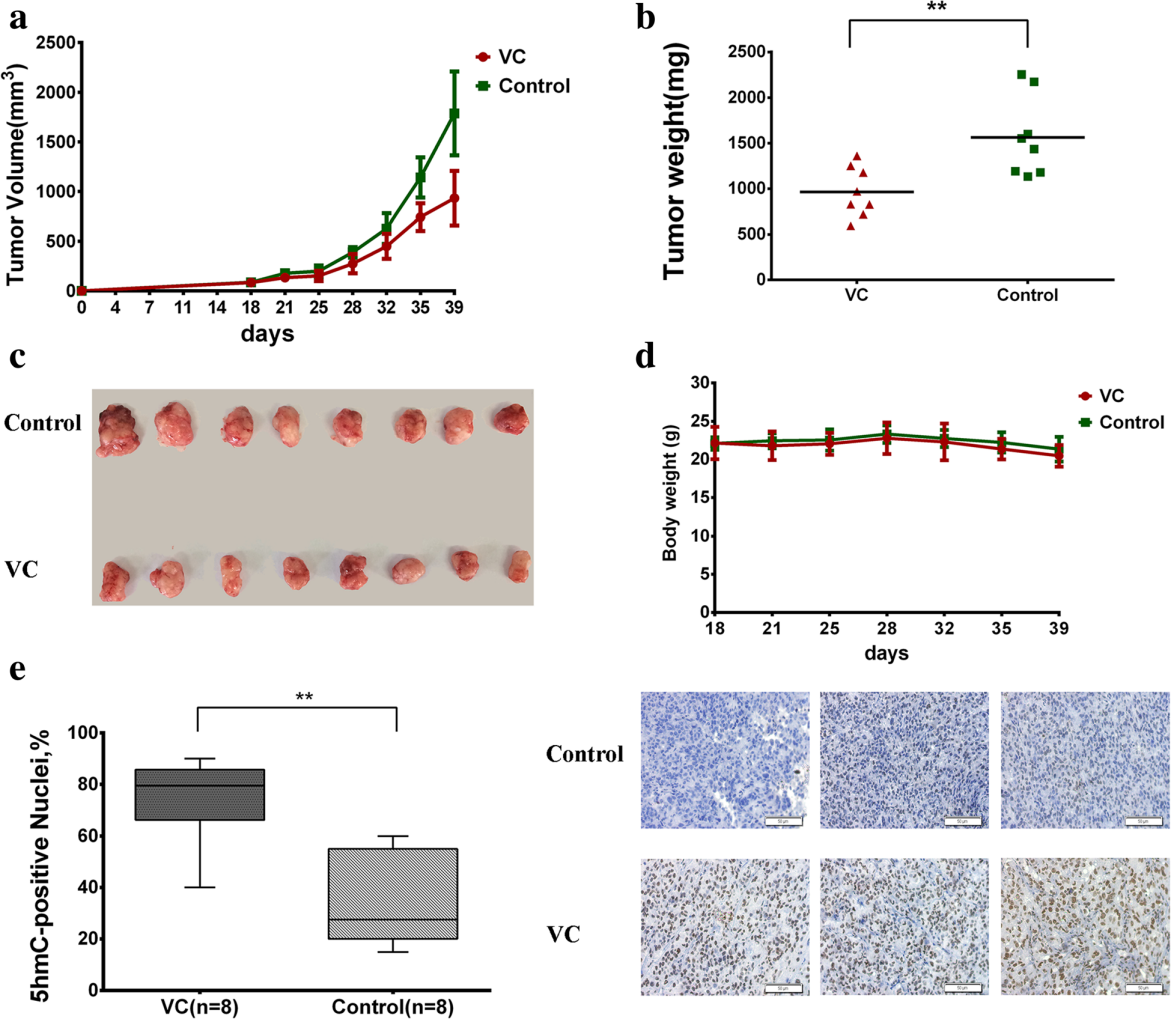
Vitamin C treatment increases 5hmC levels and decreases malignant phenotypes in T24 cells in vivo. **a** Tumor growth curves of xenografts with T24 cells treated with vitamin C or placebo. Tumor volume is shown as the mean ± SD (*n* = 8 mice). **b** Tumor weights of the indicated xenografts at the endpoint (39 days) are shown as the mean ± SD (*n* = 8 mice). Statistical significance was determined by the Mann-Whitney *U* test. **c** Images of xenograft tissues in vitamin C in treated and control groups (*n* = 8). **d** The body weights of mice during treatment (*n* = 8 mice). **e** 5hmC score and representative IHC staining of xenograft tissues. Scale bar, 50 μm. Statistical significance was determined by the Mann-Whitney *U* test

Additionally, an intraperitoneal injection of 2 g/kg/day vitamin C induced slower growth and a smaller tumor burden than saline in vivo without significant toxicity or weight loss (Fig. 4a–d). IHC staining also revealed increased 5hmC levels in the vitamin C treatment group (Fig. 4e). Collectively, our results support that vitamin C can increase 5hmC levels and block bladder cancer growth.

### Vitamin C can re-establish the 5hmC landscape in bladder cancer cells

**Fig. 5:**
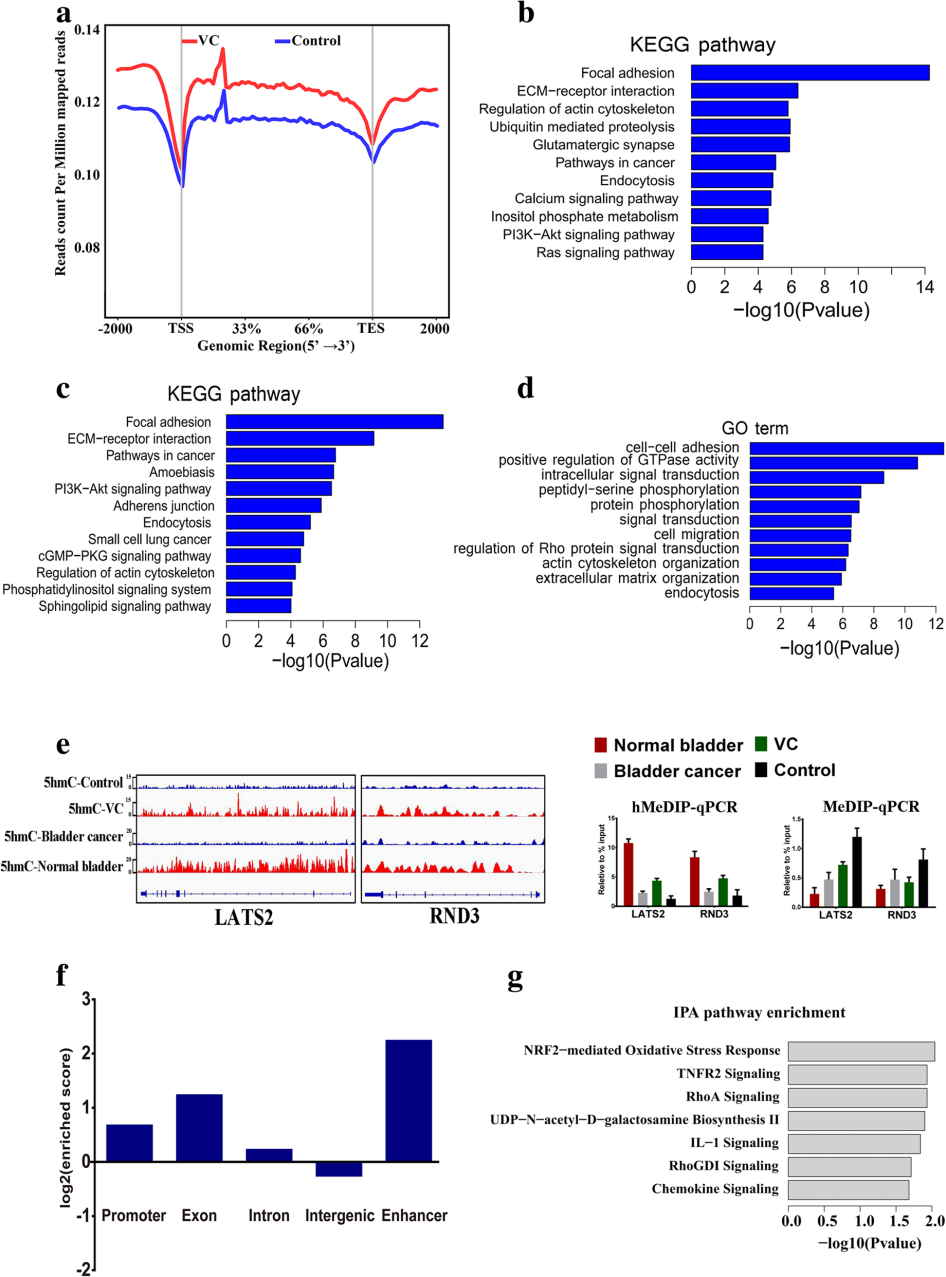
Vitamin C treatment re-establishes the 5hmC landscape in the bladder cancer cell epigenome. **a** Average 5hmC levels in T24 cells treated with vitamin C or control across different gene-associated regions. **b** KEGG pathway analysis results for significantly elevated 5hmC peak-associated genes. **c** KEGG pathway analysis results for overlapping 5hmC peak-associated genes. **d** GO term analysis results for overlapping 5hmC peak-associated genes. **e** The hMeDIP-seq results and hMeDIP-qPCR/MeDip-qPCR verifications of representative overlapping LATS2 and RND3 genes. **f** The enrichment scores of vitamin C-restored 5hmC peaks in different genomic elements. **g** IPA pathway enrichment analysis for enhancer-assigned genes

We next explored the re-established pattern of 5hmC with vitamin C treatment. T24 cells treated with 0.25 mM vitamin C or without treatment (control) were used to profile genome-wide 5hmC patterns by hMeDIP-seq. We found that vitamin C-treated bladder cancer cells showed re-establishment of the 5hmC landscape, which was similar to the landscape of normal bladder tissues (Fig. 5a). Using MACS software, 28,015 5hmC peaks and 6809 mapped genes were identified. KEGG pathway enrichment and GO term analyses of the 6809 genes revealed that these genes were closely associated with various cancer-related pathways (Fig. 5b). By overlapping the 5hmC profiles from normal tissues and vitamin C-treated T24 cells, we identified 2511 RefSeq genes with decreased 5hmC densities in bladder cancer tissue that were restored by vitamin C treatment in T24 cells. KEGG pathway and GO term analyses revealed that these genes were mainly associated with adhesion and cancer-related genes (Fig. 5c, d). As exemplified by the LATS2 and RND3 genes, vitamin C restored 5hmC levels in T24 cells; these levels were relatively low in bladder cancer cells compared with normal bladder cells, and 5mC showed an opposite trend (Fig. 5e). We further mapped these 28,015 5hmC peaks to different elements and found that the peaks were more enriched in the enhancers, exons, and promoters than in introns and intergenic regions (Fig. 5b). It was reported that 5hmC in the enhancer regions is associated with gene expression [18]. We identified 154 enhancers that overlapped with vitamin C-increased 5hmC peaks. IPA analysis of the 382 enhancer-assigned genes showed that the most significantly enriched pathways were cancer-related pathways, such as NRF2-mediated oxidative stress response, TNFR2 signaling, RhoA signaling, and IL-1 signaling.

### Vitamin C treatment shifted the transcriptome of bladder cancer cells

**Fig. 6:**
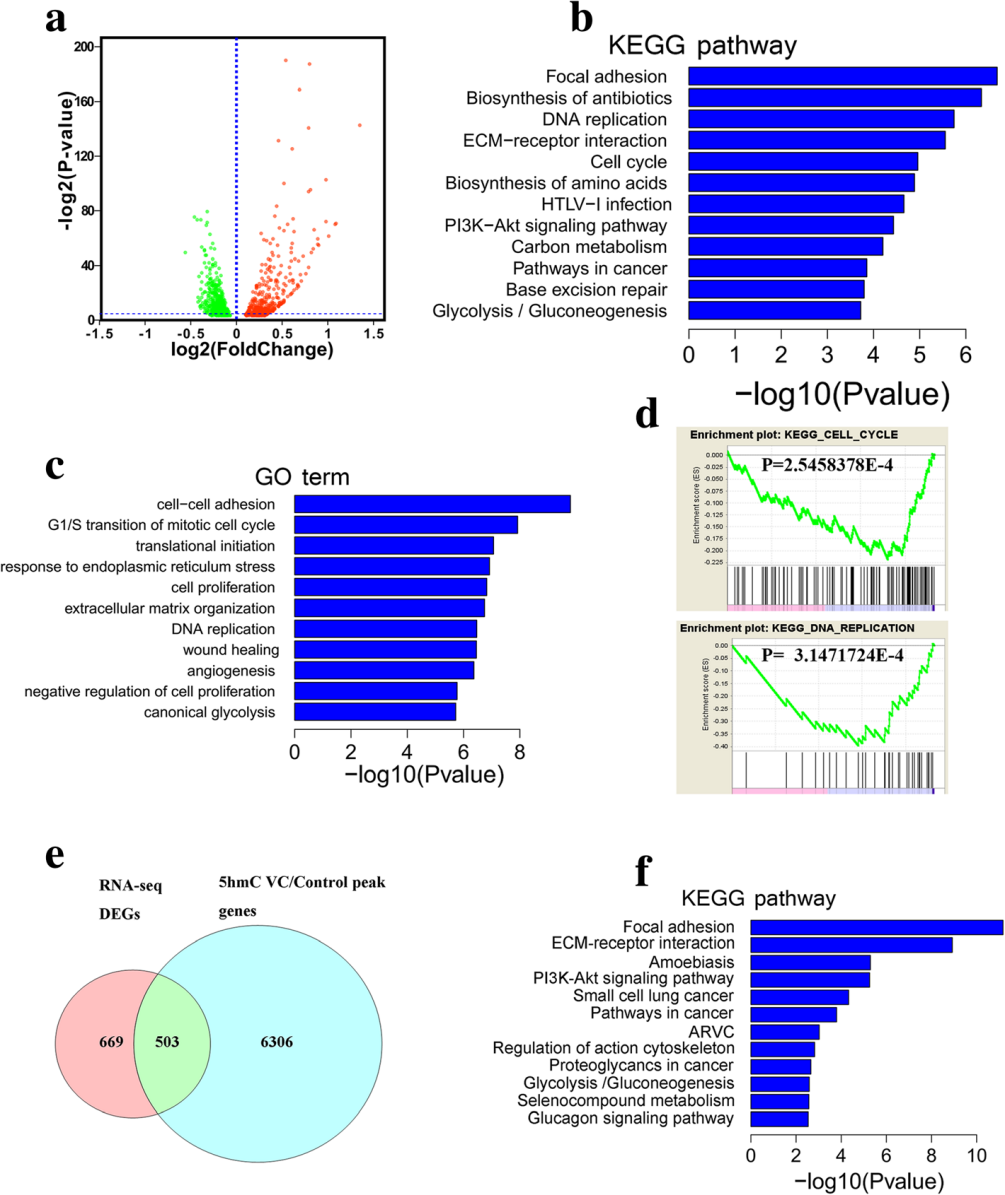
Vitamin C treatment shifted the bladder cancer cell transcriptome. **a** Volcano plot figure for differentially expressed genes treated with vitamin C. **b**, **c** KEGG pathway and GO term analysis results for differentially expressed genes, respectively. **d** Representative GSEA enrichment plots for enriched gene sets. **e** Venn diagrams showing the overlap between elevated 5hmC peak-associated genes and differentially expressed genes. **f** KEGG pathway analysis results for overlapping genes

We next examined the phenotypic changes in T24 cells at the global transcriptome level after treatment with 0.25 mM vitamin C. Using DEseq2, 1172 differentially expressed genes were identified, including 482 upregulated and 690 downregulated genes (Fig. 6a and Additional file 3: Table S1). KEGG pathway and GO term analyses showed that these genes were mainly associated with focal adhesion, DNA replication, cell cycle, and several cancer-related pathways (Fig. 6b, c and Additional file 4: Table S2). Gene set enrichment analysis (GSEA) analysis also showed an inhibitory effect on the cell cycle and DNA replication (Fig. 6d). Furthermore, among the 1172 genes, 503 genes overlapped with the 6809 restored 5hmC-related genes (Fig. 6e). KEGG pathway analysis of these 503 genes also showed enriched focal adhesion and several cancer-related pathways (Fig. 6f). We overlapped these 1172 genes with the genes that showed decreased 5hmC in bladder cancer tissue, and 499 genes were identified as common to both sets. KEGG pathway analysis of these 499 genes also showed enriched focal adhesion and several cancer-related pathways (Additional file 2: Figure S2D). Furthermore, we intersected three sets of genes (genes with decreased 5hmC in bladder cancer tissue, genes with increased 5hmC after vitamin C treatment, and 1172 differently expressed genes) and found 265 overlapping genes. KEGG pathway analysis of these 265 genes showed enriched focal adhesion and several cancer-related pathways (Additional file 2: Figure S2E). Therefore, vitamin C treatment shifted the transcriptome of bladder cancer cells to inhibit malignant phenotypes.

## Discussion

In this study, we illustrated the genome-wide profile of 5hmC loss in bladder cancer and the prognostic value of global 5hmC levels. Furthermore, we found that vitamin C increased 5hmC content and inhibited the malignant phenotypes associated with bladder cancer. Our results showed that the loss of 5hmC and 5hmC restoration by vitamin C were significantly associated with cancer-related genes.

5hmC was first discovered in the 1970s and has received renewed attention in the last decade for its involvement in the regulation of embryonic stem (ES) cell differentiation and nervous system development [19, 20]. The loss of 5hmC has been identified as a novel hallmark in most cancers [9–12]. In contrast to 5mC, 5hmC was found to be an important epigenetic mark of active genes and was mainly enriched in gene bodies [11]. These findings indicate that 5hmC might play an important role in tumorigenesis and progression. Various mechanisms may underlie 5hmC depletion in cancer, such as mutations in TETs and IDHs, which thus decrease the expression of TETs and IDHs [12, 21, 22]. Tumor hypoxia is also thought to be a reason for the loss of 5hmC by reducing TET activity [23]. Restoring 5hmC levels by overexpressing TET2 in melanoma cells, TET1 in breast cancer cells, and IDH1 in renal cancer cells demonstrated inhibitory effects in vitro and in vivo [11, 12, 24].

The inhibitory effects of vitamin C both in vitro and in vivo have been reported in several cancers [25–28]. A number of clinical experiments have been conducted in advanced cancer patients, and they revealed that vitamin C is a safe and well-tolerated micronutrient that can inhibit tumors [25, 26, 29]; however, the underlying therapeutic mechanism has remained largely undefined. One study suggested that vitamin C could inhibit nonsmall cell lung cancer (NSCLC) and glioblastoma (GBM) cells by increasing the levels of H_2_O_2_ and disrupting intracellular iron metabolism [26]. Other studies recently showed that vitamin C selectively killed KRAS and BRAF mutant colorectal cancer cells by targeting glyceraldehyde-3-phosphate dehydrogenase (GAPDH) [30].

As a cofactor, vitamin C can enhance the activity of Fe (II)-2-oxoglutarate dioxygenases, including TETs, leading to DNA demethylation [31]. Recent studies have shown that the suppression effects of vitamin C partly result from the demethylation caused by activating TET. In leukemia, vitamin C suppressed hematopoietic stem cell (HSC) frequency and leukemogenesis by promoting TET activity [16, 17]. Vitamin C restored 5hmC levels in melanoma and rebuilt the transcriptome in melanoma cells [28]. Another study also showed that vitamin C increased TET activity, leading to DNA demethylation in lymphoma cells independently of hydrogen peroxide [32]. Vitamin C also enhanced the antitumor effect of standard therapies. Vitamin C abrogated cetuximab resistance mediated by mutant KRAS in human colon cancer cells [33]. Another study reported that vitamin C enhanced the chemosensitivity of ovarian cancer and reduced the toxicity of chemotherapy [34]. Even at the physiological level, vitamin C also enhanced the effects of decitabine (5-aza-2′-deoxycytidine) in colorectal carcinoma, acute myeloid leukemia, breast carcinoma, and hepatocellular carcinoma cells [35]. Consistent with previous studies, we found that high-dose vitamin C could directly cause selective bladder cancer cell death and apoptosis in an H_2_O_2_-dependent manner and that low-dose vitamin C suppressed bladder cancer cell growth in an H_2_O_2_-independent manner that included 5hmC restoration.

In this study, we analyzed the transcriptome of bladder cancer cells after vitamin C treatment. However, the results of RNA-seq and hMeDip-seq did not fit very well. A total of 482 genes were upregulated, 690 genes were downregulated following vitamin C treatment, and 503 genes correlated with changes in 5hmC levels. The mechanisms of how these 5hmC changes alter gene expression and contribute to the decreased malignancy of bladder cancer cells remain unclear and require further examination in the future. We also found that the IC50 of vitamin C for different types of cancers was variable. In this study, the IC50 of vitamin C for bladder cancer was 0.5 mM, and the IC50 for renal cancer cells was above 1 mM. In a study by Christopher B Gustafson, the IC50 of vitamin C for melanoma cells was approximately 0.5 mM [28]. Chen et al. compared the cytotoxicity of vitamin C on different cancer cells and found that different cell lines have various sensitivities [25]. The underlying mechanism of the inhibitory effect of vitamin C on different types of cancers requires additional study.

## Conclusions

Our results suggest that the loss of 5hmC is a novel hallmark of bladder cancer with prognostic and outcome significance. We also demonstrated that vitamin C treatment can decrease the malignant phenotypes of bladder cancer in vitro and in vivo by partially increasing the global content of 5hmC and consequently altering the transcriptome. These results suggest that vitamin C could be a potential epigenetic treatment for bladder cancer and perhaps other types of cancer.

## Methods

### Cell culture and treatment

SV-HUC-1 normal human urinary epithelial cells were obtained from ATCC and cultured in F-12K medium (Gibco, USA). Human normal bladder primary epithelial cells Hum-u007 (obtained from iCell Bioscience Inc., Shanghai) were cultured in n ICell Primary Keratinocyte Culture System (PriMed-iCell-010). Bladder cancer cell lines T24 and 5637 cells were cultured in RPMI-1640 medium (Gibco, USA), and UMUC-3 and J82 cells were cultured in MEM medium (Gibco, USA). All bladder cancer cell lines were obtained from the Institute of Urology at Peking University (Beijing, China). All media contained 10% fetal bovine serum (Gemini, USA), penicillin G (100 U/ml), and streptomycin (100 μg/ml) (Sigma-Aldrich, Germany). Cells were maintained as a monolayer culture at 37 °C in a humidified atmosphere containing 5% CO_2_. After seeding on plates for 24 h, the cells were treated with vitamin C (l-ascorbic acid, A4034, Sigma-Aldrich, St Louis, MO, USA) at different concentrations for varying durations. Catalase was obtained from Sigma (C1345).

### Immunohistochemistry

Surgical specimens were obtained from 135 patients diagnosed with bladder urothelial carcinoma at the Department of Urology, Peking University First Hospital, between 2010 and 2015. All patients signed informed consent, and the ethics committees of the Peking University First Hospital approved the protocol.

After fixing the tissues with 4% formalin and embedding them in paraffin wax, the tissues were cut into 5-μm sections using a microtome. The sections were deparaffinized in xylene and rehydrated with graded concentrations of alcohol. Subsequently, the slides were treated with 3% H_2_O_2_ to block endogenous peroxidase activity and were heated (95 °C) for 2.5 min in citrate buffer (10 mmol/l, pH 6.0) for antigen retrieval. To reduce nonspecific binding, 10% normal goat serum was applied. Subsequently, the slides were incubated with anti-5hmC antibody (Active Motif, USA, 39769, 1:10,000) at 4 °C overnight. A PowerVision™ two-step histostaining reagent and 3,3-diaminobenzidine tetrahydrochloride substrate kit (ZSGB-Bio, China) were used to visualize the localization of the antigen according to the manufacturer’s instructions.

The staining score of the 5hmC in tissues was evaluated by two independent pathologists by counting 5hmC-positive nuclei at 0~10%, 11~30%, 31~50%, and > 50% levels, and a positive rate ≥ 31% was defined as a high 5hmC level.

### Dot blot

Genomic DNA was extracted from cultured cells using QIAamp DNA Mini Kits (Qiagen, Germany) according to the manufacturer’s instructions. A Qubit Fluorometer (Life Technology, USA) was used to quantify the DNA concentration. DNA samples were diluted with 2 N NaOH and 10 mM Tris·Cl at pH 8.5 and then loaded onto a Hybond N+ nylon membrane (GE Health, USA) using a 96-well dot blot apparatus (Bio-Rad, USA). After baking at 80 °C for 60 min and being blocked with 5% nonfat milk for 1 h at room temperature, the membrane was incubated in anti-5hmC (Active Motif, 39769) and anti-5mC antibodies (ZYMO RESEARCH, #A3001-200) at 4 °C overnight and visualized by chemiluminescence. To ensure equal loading, the membrane was then stained with methylene blue.

### hMeDIP-seq

One pair of bladder urothelial carcinoma and relative normal bladder tissues was used for hMeDip-seq. The sequencing libraries were prepared with 10 μg of genomic DNA ligated to PE adaptors (Illumina, USA), which was followed by 5hmC antibody capture for immunoprecipitation. The hydroxymethylated fragments were amplified with 10–12 cycles using adaptor-specific primers (Illumina, USA) and quantified on an Agilent 2100 Bioanalyser before cluster generation and sequencing on a HiSeq 3000 according to the manufacturer’s protocols.

### Identification of vitamin C-restored 5hmC peaks

Briefly, the reads were aligned to the hg19 human genome (bowtie2, default parameters) and de-duplicated; unique reads were kept. Significantly, enriched regions were determined using model-based analysis with the ChIP-Seq (MACS) package (v.2.1.0, default settings). GO term and KEGG pathway analyses were performed with the Database for Annotation, Visualization, and Integrated Discovery (DAVID) program.

### Definition of enhancers

The enhancers in adult normal bladder tissue were identified using Roadmap H3K27ac CHIP-seq data (GSM1013133 and GSM1059457). The enhancer was assigned to the nearest gene within a distance of ~ 50 kb [36].

### RT-qPCR

Total RNA was isolated from cell lines using the TRIzol reagent (Invitrogen, Thermo Fisher Scientific, Inc.). A total of 2 μg of RNA was reverse-transcribed into cDNA using M-MLV reverse transcriptase (Promega, USA) and oligo (dT) 15 (Promega, USA) as a primer. Quantitative PCR was performed using SYBR® FAST qPCR Kits (KAPA Biosystems, USA) in a final volume of 10 μl in the 7500 Fast Real-Time PCR System (Applied Biosystems, Thermo Fisher Scientific, Inc.). All primer pairs are shown in Table 3, and glyceraldehyde-3-phosphate dehydrogenase (GAPDH) served as the endogenous control. The expression of target mRNA was normalized to GAPDH according to the ΔΔCq method.

**Table 3:** Primer pairs for real-time PCR

Genes	Sequence (5′ to 3′)	
GAPDH	Sense	GGTGAAGGTCGGAGTCAACG
	Antisense	TGGGTGGAATCATATTGGAACA
TET1	Sense	AATGGAAGCACTGTGGTTTG
	Antisense	ACATGGAGCTGCTCATCTTG
TET2	Sense	AATGGCAGCACATTGGTATG
	Antisense	AGCTTCCACACTCCCAAACT
TET3	Sense	GAGGAGCGGTATGGAGAGAA
	Antisense	AGTAGCTTCTCCTCCAGCGT
SVCT1	Sense	TCATCCTCCTCTCCCAGTACCT
	Antisense	AGAGCAGCCACACGGTCAT
SVCT2	Sense	TCTTTGTGCTTGGATTTTCGAT
	Antisense	ACGTTCAACACTTGATCGATTC
IDH1	Sense	TCCGTCACTTGGTGTGTAGG
	Antisense	GGCTTGTGAGTGGATGGGTA
IDH2	Sense	TGAACTGCCAGATAATACGGG
	Antisense	CTGACAGCCCCCACCTC
L2HGDH	Sense	TCAAAAATTCATCCCTGAAATTACT
	Antisense	CTACCAGATTTCCATCTCTATCCAG

### MTS cell viability assay, apoptosis assay, and colony formation assay

Cell viability assays were assessed using the CellTiter 96® AQ One Solution Reagent (Promega, USA) according to the manufacturer’s instructions. An apoptosis assay was performed by FACS with Annexin V-FITC/PI staining. For colony formation assays, cells were seeded in 6-well plates at a density of 800 cells/well and cultured with different concentrations of vitamin C at 37 °C for 7 days.

### Western blot analysis

Total protein of cell lines was prepared with ice-cold radioimmunoprecipitation assay buffer (Sigma-Aldrich; Merck Millipore) and quantified using BCA protein assay reagent (Pierce Chemical Co., Rockford, IL, USA). Equal amounts of proteins were separated by SDS-PAGE and transferred to a polyvinylidene fluoride membrane. After blocking for 1 h with 5% nonfat milk, the membranes were incubated overnight at 4 °C with Caspase-3 (Abcam, ab13847), Bcl-2 (Abcam, ab32124), Bcl-xl (Abcam, ab32370), PARP (Abcam, ab191217), and beta-actin (Proteintech, 66009). After washing and incubating the membranes with secondary antibodies, signals were detected by applying ECL Western Blotting Detection Reagent (GE Healthcare Life Sciences).

### Mice xenograft model

T24 cells (1 × 10^6^ cells) were resuspended in 100 ml of PBS and subcutaneously injected into the axillary fossae in 4-week-old nude mice (BALB/c-nu). Tumor volume was calculated with the formula *V* = 0.5 *ab*^2^, where *a* is the longest tumor axis, and *b* is the shortest tumor axis. When the volume of the tumors reached approximately 150 mm^3^, mice with tumors were intraperitoneally injected with vitamin C (2 g/kg/day) or saline. The animal protocol was approved by the animal ethics committee of Peking University First Hospital.

### RNA-seq and gene set enrichment analysis

The KAPA Stranded RNA-seq Library Preparation Kit was used to construct RNA-seq libraries according to the manufacturer’s instructions. Sequencing reads were aligned to the hg19 human genome using the Tophat program (tophat v2.1.1) with the default parameters. Total read counts for each protein-coding gene were extracted using HTSeq (HTSeq version 0.6.0) and then loaded into the R-package DEseq2 to calculate the differentially expressed genes with FDR < 0.05. GSEA was performed using C2 (curated gene sets) collections.

### Statistical analysis

All data were evaluated with SPSS version 22.0 software (SPSS, Chicago, IL, USA). The Kaplan-Meier method and corresponding log-rank test were performed to determine the differences in postoperative survival rates. For univariate and multivariate analysis, the Cox regression method was used. Statistical significance was defined as *P < 0.05 and **P < 0.01.

## Additional files

Additional file 1: **Figure S1.** (A) 5hmC level scoring by IHC staining (0–10%, 11–30%, 30–50%, and > 50%). (B) Dot blot assay of 5hmC levels of J82 and 5637 cells at varying vitamin C concentrations. (C) Dot blot assay of 5mC levels of J82 and 5637 cells at varying vitamin C concentrations. (D) Dot blot assay of 5hmC levels of 786-O and A498 cells and MTS assay of cell viability at varying vitamin C concentrations. (TIF 3349 kb)

Additional file 2: **Figure S2.** (A) The relative transcription levels measured by RT-qPCR of TET1/2/3 in T24 cells treated with or without vitamin C. Statistical significance was determined by the Mann-Whitney *U* test. (B) Western blot of apoptosis markers for T24 cells at varying vitamin C concentrations. (C) Cell viability measured with MTS of T24 and 5637 cells at varying vitamin C concentrations with or without catalase at 100 μg/ml. Statistical significance was determined by the Mann-Whitney *U* test. (D) Venn diagrams showing the overlap between decreased 5hmC peak-associated genes in bladder cancer and differentially expressed genes (left); KEGG pathway analysis results for overlapping genes (right). (E) Venn diagrams showing the overlap between decreased 5hmC peak-associated genes in bladder cancer; increased 5hmC peak-associated genes after vitamin C treatment, and differentially expressed genes (left); KEGG pathway analysis results for overlapping genes (right). (TIF 980 kb)

Additional file 3: **Table S1.** List of differential expression genes in vitamin C treated and control T24 cells. (XLSX 224 kb)

Additional file 4: **Table S2.** KEGG_PATHWAY analysis of 1172 differential expression genes in Fig. 6b. (XLSX 78 kb)

## Abbreviations

5hmC: 5-Hydroxymethylcytosine
5mC: 5-Methylcytosine
hMeDIP: Hydroxymethylated DNA immunoprecipitation
IDH: Isocitrate dehydrogenases
IHC: Immunohistochemical staining
ITIH5: Inter-alpha-trypsin inhibitor heavy chain family member 5
LATS2: Large tumor suppressor kinase 2
MeDIP: Methylated DNA immunoprecipitation
RND3: Rho family GTPase 3
SVCT: Sodium-dependent vitamin C transporters
TET: Ten-eleven translocation family dioxygenases
TIMP2: TIMP metallopeptidase inhibitor 2
