# Supplementary figures and images for: Vitamin C increases 5-hydroxymethylcytosine level and inhibits the growth of bladder cancer
Source: Clin Epigenetics. 2018 Jul 13;10:94. doi: 10.1186/s13148-018-0527-7 (PMC6045833; doi:10.1186/s13148-018-0527-7)

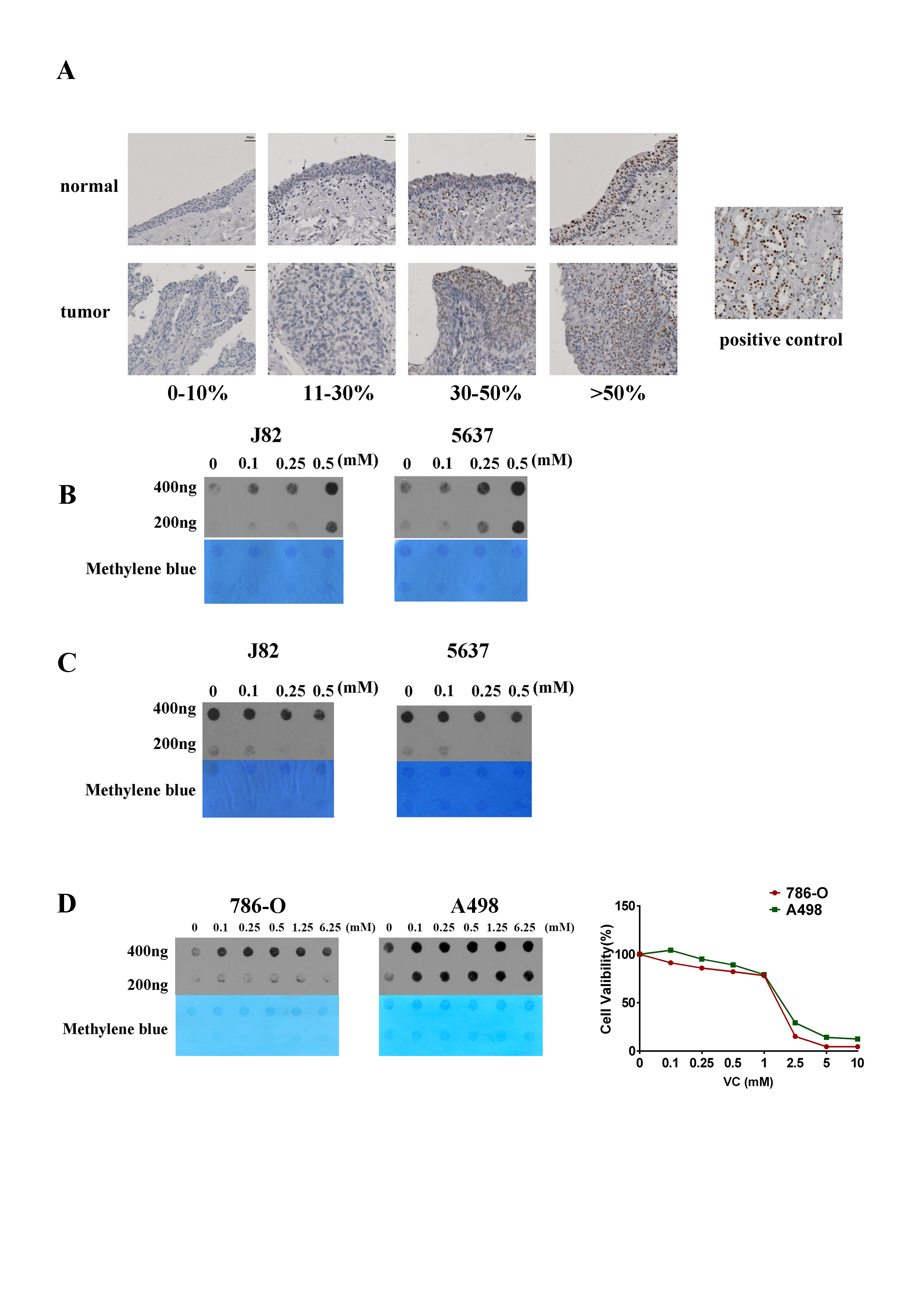

Supplement: Supplementary file 1 — Figure S1. (A) 5hmC level scoring by IHC staining (0–10%, 11–30%, 30–50%, and > 50%). (B) Dot blot assay of 5hmC levels of J82 and 5637 cells at varying vitamin C concentrations. (C) Dot blot assay of 5mC levels of J82 and 5637 cells at varying vitamin C concentrations. (D) Dot blot assay of 5hmC levels of 786-O and A498 cells and MTS assay of cell viability at varying vitamin C concentrations. (TIF 3349 kb) [file 13148_2018_527_MOESM1_ESM.tif]

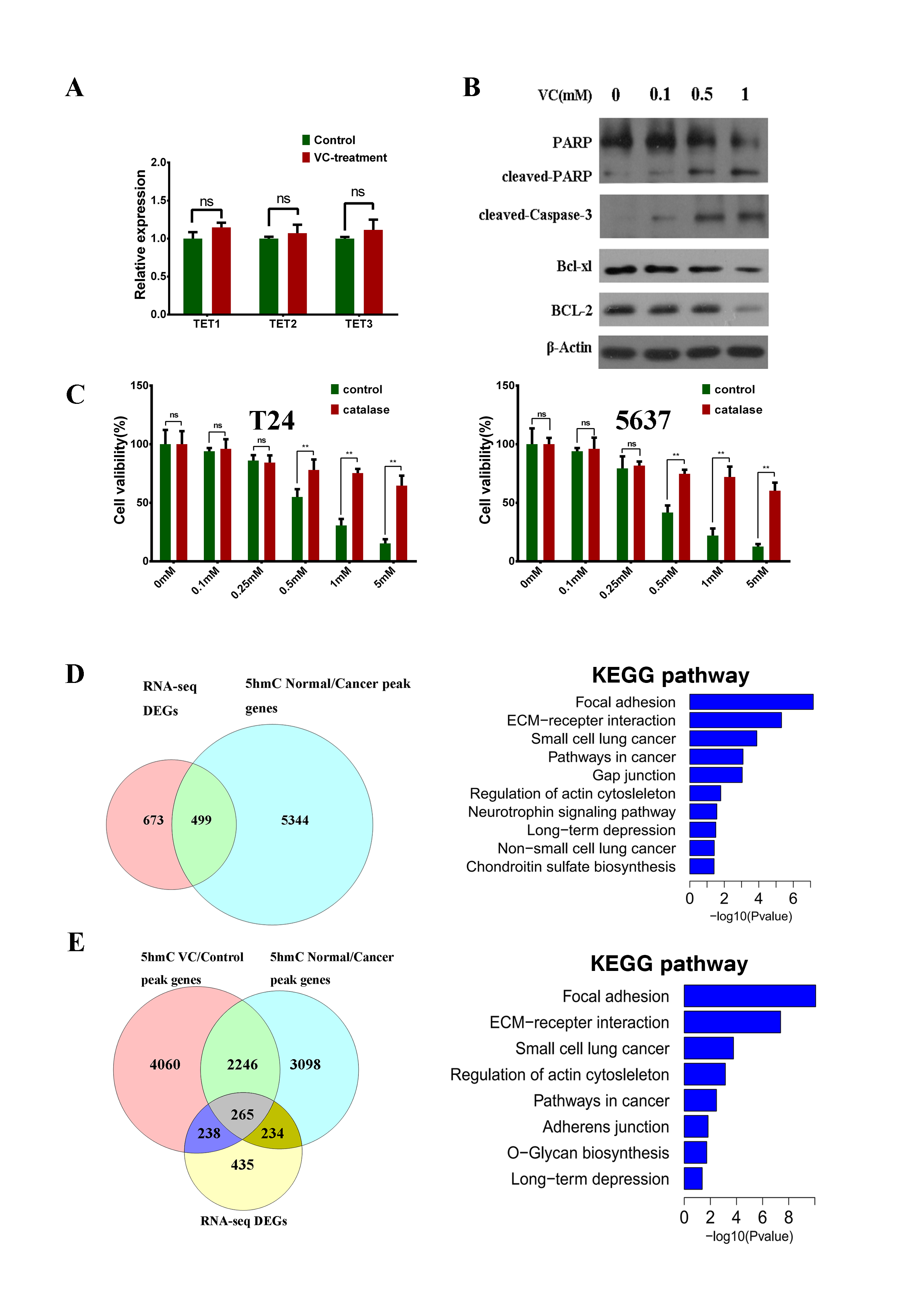

Supplement: Supplementary file 2 — Figure S2. (A) The relative transcription levels measured by RT-qPCR of TET1/2/3 in T24 cells treated with or without vitamin C. Statistical significance was determined by the Mann-Whitney U test. (B) Western blot of apoptosis markers for T24 cells at varying vitamin C concentrations. (C) Cell viability measured with MTS of T24 and 5637 cells at varying vitamin C concentrations with or without catalase at 100 μg/ml. Statistical significance was determined by the Mann-Whitney U test. (D) Venn diagrams showing the overlap between decreased 5hmC peak-associated genes in bladder cancer and differentially expressed genes (left); KEGG pathway analysis results for overlapping genes (right). (E) Venn diagrams showing the overlap between decreased 5hmC peak-associated genes in bladder cancer; increased 5hmC peak-associated genes after vitamin C treatment, and differentially expressed genes (left); KEGG pathway analysis results for overlapping genes (right). (TIF 980 kb) [file 13148_2018_527_MOESM2_ESM.tif]
